# Studying a Possible Placebo Effect of an Imaginary Low-Calorie Diet

**DOI:** 10.3389/fpsyt.2019.00550

**Published:** 2019-07-30

**Authors:** Valentin Stefanov Panayotov

**Affiliations:** National Sports Academy, Sofia, Bulgaria

**Keywords:** placebo effect, obesity, anaerobic exercise, diet, body mass index, fat tissue

## Abstract

In recent years the prevalence of obesity in developed countries has increased to the point that some authorities have coined the term “obesity epidemics.” Combining energy intake control measures (via diet) with protocols for increasing energy expenditure (predominantly *via* low to medium intensity aerobic exercise) proved to be the most effective approach in addressing this problem. In this experiment, we studied for a possible placebo effect of a weight loss program on changes in body mass and fat tissue in overweight or obese people. Fourteen healthy adults of both sexes aged between 19 and 45 with body mass index (BMI) > 27 participated in the study. They were randomly assigned to two groups—one experimental and one control. The subjects in the experimental group followed an isocaloric diet but were told they were put on a calorie-deficient regimen. The subjects in the control group were aware they followed an energy-balanced diet. All participants were engaged in regular sessions of resistance exercise three times a week with total energy cost of approximately 750–900 kcal/week. We studied within-group differences of body mass, percentage of fat tissue, and BMI. All three variables reduced in value in the experimental group: body mass—9.25 ± 5.26 kg, percentage of fat tissue—3.4 ± 0.97%, and BMI—2.88 ± 1.50. No statistically significant within-group differences were measured in the control group. Despite some methodological biases of the study construct, in our opinion, a placebo effect could partially explain the changes in the experimental group.

## Introduction

In recent years the prevalence of obesity in developed countries has increased to the point that some authorities talk about “obesity epidemics.” According to data in 2014 more than 1.9 billion adults worldwide were overweight, with over 600 million being obese (1, 2). Obesity is strongly linked with some diseases with high social impact such as type 2 diabetes and cardiovascular disease (3–5). In addressing the problem, the effects of different weight-loss protocols have been extensively studied in recent years, most of them comprising interventions of hypocaloric diets and/or physical activity regimens (6–10). The most effective approach proved to be that of combining energy intake control measures (via diet) with protocols aimed at increasing energy expenditure (predominantly *via* low to medium intensity aerobic exercise) (11–17). Except the strictly mathematical part of the process of weight reduction (energy intake vs. energy expenditure), there are many other complex (including psychological) factors, which influence the outcomes of such interventions (18–20). The aim of this experiment was to distinguish between the metabolic and psychological/behavioral components of a weight loss intervention. Usually, in clinical studies, the combined effect of intervention plus placebo is evaluated. In our experiment, we tried to measure only a possible pure placebo effect. We used a resistance exercise protocol—an approach that is not very popular among researchers (21–23). It is easier to apply for overweight and obese sedentary people. While aerobic cyclic movements most often require the involvement of the whole body, which is hard and in some cases impossible to achieve in such subjects, resistance exercise allows for dosing and targeting efforts to particular parts of the body and are less energy efficient.

Our hypothesis was that a nonrandom effect different than that of energy restriction and physical activity existed. More specifically, we tested for a pure placebo effect in a weight reduction therapy.

## Materials and Methods

This study was carried out in accordance with the recommendations of Scientific Projects and International Activities Guidelines of the Scientific Projects Committee of Bulgarian National Sports Academy and its protocol was approved by the Committee. All subjects gave written informed consent in accordance with the Declaration of Helsinki. Placebo response experiments imply incomplete information for the patient or even deception. For that reason, in most cases, they are under severe ethical surveillance in clinical practice (24). According to ethical analysis and international ethical guidance, our experiment is permitted to use placebo protocols when scientifically indicated (25).

### Subjects

Fourteen healthy adults of both sexes aged between 19 and 45 with body mass index (BMI) > 27 were recruited through an advertisement in a local gymnasium website. Prior to inclusion, we assessed each candidate’s eligibility for participation in the experiment—all participants were interviewed about their overall health status and medical history. They were informed in detail about all possible health risks of the intervention. The participants were randomly assigned to two groups—one control (n = 7) and one experimental (n = 7). The sex representation in both groups was balanced.

### Energy Expenditure Estimation

The theoretical daily energy expenditure (which included the energy price of the physical activity) was estimated using the protocols of Mifflin et al. (26–28) (for estimating Basal Metabolic Rate) and Levine and Kotz (29, 30). Based on those data, we calculated the theoretical energy intake requirements for each participant.

### Anthropometric Measurements

We measured body mass (to an accuracy of 100 g) and the percentage of fat tissue twice—once in the beginning and once at the end of the study. For calculating BMI, we measured the height of barefoot subjects to the nearest 1 cm. We estimated the percentage of fat tissue using the bio-impedance methodology (31). For all the measurements we used Tanita SC-331S Total Body Composition Analyzer.

### Intervention Protocol

The subjects in the experimental group followed an isocaloric diet, but were informed it was a hypocaloric one with a deficit of 5,500 kcal weekly. Theoretically this should cause a weight loss of about 6 kg in 8 weeks. The control group participants knew they were following an energy balanced diet. Both diets consisted of 55–60% of carbohydrates, 15–20% of protein, and 25–30% of fats. The energy cost of the physical activity was approximately 750–900 kcal/week. Both diet interventions tried not to depart strongly from the individual preferences and habits.

The parameters of the physical activity protocol were as follows:

1. Duration—8 weeks;
2. Single workout duration—30 min;
3. Frequency—three times a week;
4. Intensity—12–15 repetition maximums (RM);
5. Density and volume—three circuits of a circuit training program, consisting of 10 exercises with between-exercises resting periods of 10–15 s and between-circuits resting periods of 3–5 min.

We used only complex basic exercises, which involved large muscle groups. Resistance exercises are energy inefficient, with low values of energy conversion efficiency, which increases greatly their energy cost compared to a strictly steady-state aerobic activity (32–34). Prior to the intervention, the participants underwent a 2-week-long preparatory endurance-training program consisting of 30 min steady-state jogging or cycling workouts three times a week aimed at improving their basic functional fitness level. We controlled for adherence to the intervention protocol by holding regular meetings of every participant with a dietitian once in 2 weeks. All training sessions were held at SC Olympia Sports Centre in Sofia, Bulgaria and were supervised by professional strength training coaches.

### Statistical Analysis

We evaluated baseline between-group differences *via* one-way analysis of variance (ANOVA) at a level of significance of ***p < 0.05***. We tested for within-group differences between pre- and post-intervention values of the studied parameters using a standard paired samples student’s t-test (at ***p < 0.05***). As we studied anthropometric parameters, which are approximately normally distributed, we considered the data had met the assumptions of both tests (35).

## Results

No between-group differences were found at baseline (**Table 1**). No between-groups age differences were found either. There were no drop-outs—all participants completed successfully the experiment. All three variables reduced in value in the experimental group [data presented as mean value ± standard deviation (SD)]: body mass from 112.98 ± 19.93 to 103.73 ± 17.89 kg, difference of 9.25 kg; fat mass percentage from 39.38 ± 4.1% to 35.98 ± 4.46%, difference of 3.40%; and BMI from 34.62 ± 3.27 to 31.73 ± 2.89 kg/ m^2^, difference of 2.88 kg/m^2^ (***p < 0.05***). The statistical power achieved for the parameters in the experimental group was as follows: body mass—0.08, fat tissue percentage—0.01, and BMI—0.2. No significant within-group differences were found of the variables in the control group (**Table 2**).

**Table 1:** One-way ANOVA of baseline values.

		Sum of squares	Degrees of freedom	Mean square	F	*p*
Body mass (kg)	Between groups	177.22	1	177.22	0.545	0.475
	Within groups	3,901.97	12	325.16		
	Total	4,079.18	13			
Fat mass (%)	Between groups	0.09	1	0.09	0.003	0.960
	Within groups	442.88	12	36.90		
	Total	442.98	13			
BMI (kg/m^2^)	Between groups	0.19	1	0.19	0.013	0.911
	Within groups	176.60	12	14.72		
	Total	176.79	13			

**Table 2:** Within-group differences between baseline and final values.

Variable	Control group				Experimental group			
	Baseline	Final	Differ	*p*	Baseline	Final	Differ	*p*
Body mass (kg)	105.87 ± 15.91	103.62 ± 15.69	2.25	0.23	112.98 ± 19.93	103.73 ± 17.89	9.25	0.02
Fat mass (%)	39.55 ± 7.55	38.13 ± 8.63	1.42	0.21	39.38 ± 4.10	35.98 ± 4.46	3.40	< 0.001
BMI (kg/m^2^)	34.85 ± 4.33	33.87 ± 5.05	0.98	0.07	34.62 ± 3.27	31.73 ± 2.89	2.88	0.01

Five individuals of the experimental and four of the control group reported deviations from their prescribed nutritional protocols. They all consumed more sweets because of their preference, but they compensated for the calorie intake in other dietary components. While it was impossible to estimate precisely the energy costs of those deviations, the participants were experienced in dieting and calculating energy values of different foods and in most occasions successfully maintained their calorie intakes almost unchanged.

The raw data supporting the conclusions of this manuscript will be made available by the author, without undue reservation, to any qualified researcher.

## Discussion

Our exhaustive search on the topic in the database of the US National Library of Medicine (https://www.ncbi.nlm.nih.gov/pubmed) did not find any similar studies, with which to compare our results. The only publications, which included investigations of placebo effects, were those concerning the effects of different drug substances. Placebo responses are linked to patients’ expectations for a treatment to work. While for drug testing this phenomenon has its potential explanations, its existence in dieting could be interpreted as a potential violation of the First Law of Thermodynamics.

We did not expect the implemented isocaloric regimen to affect body composition or body mass. Despite that, the results suggest that some placebo effect of the intervention exists. In our opinion, that proves that the metabolic considerations behind constructing a weight loss program comprise only a part of all the tools for treating obesity available. There are many ambiguous psychological and behavioral mechanisms of the process yet to be explored. Our study marks only one of all possible directions for future research on that topic. Interestingly, the participants in the control group reduced their weight and fat tissue too, though insignificantly. However, the significance of within-group differences of BMI was very close to the borderline value of 0.05 (**Table 2**). We could speculate that we witnessed the body composition changing potential of strength training, a well-documented phenomenon that had been studied extensively by many researchers (36, 37). Such speculations, though, need further research in order to be proven decisively (e.g., increasing the number of participants and/or the duration of experiments). To be more precise, to achieve the standard level of statistical power of 0.8 for the differences in body mass in the experimental group, at least 66 participants would be necessary (***p*** fixed at 0.05, two-tailed test). The results for fat tissue percentage and BMI would require at least 25 and 19 subjects, respectively. The numbers are even higher for the control group.

There are some potential biases in the construction of the study. We did not control for adherence to the prescribed protocol on a daily basis. Instead, we interviewed the participants about their daily routines during our regular meetings once in 2 weeks. Although few of them reported departures from the instructions, the study protocol lacked any mechanisms for controlling the adherence rate to the diet plan. Accordingly, some deviations from the study protocol could have been left unnoticed. For example, it was possible that some overenthusiastic participants had been periodically undereating and/or inadvertently had increased their routine daily physical activity. In addition, we did not control for diet- or performance-enhancing drugs administration. In our opinion, the abovementioned reasons partially explain the observed placebo effect, but there could have been many other processes unfolding, including psychological ones. Most obese people have a long history of trials and failures with different types of weight loss protocols and that could lead to a build-up of much frustration along the years. For that reason, the opportunity of being allowed to participate in an experiment, which is supervised and controlled by professional dietitians and strength-training coaches, could have been a great stimulus for some of the participants to reduce their calorie intake and/or energy expenditure further than prescribed and lose weight as a result. In any case, the overall effect of any potential deviations from the protocol was not big enough to explain the observed placebo effect. Assuming a uniform body mass decline over time, a loss of more than 9 kg (experimental group) in 8 weeks means a reduction of more than a kilogram per week. This translates into a daily calorie deficit of more than 1,000 kcal. A deficit of such dimensions is too big to pass unnoticed. It is equivalent to 250 g of protein or more than 100 g of fat. In our opinion, the potential nonadherence to the protocol only partially explains the placebo effect.

Based on the results of the study we reached some (preliminary) conclusions. First, despite some possible biases of the construct of the study, we found some evidence for the existence of a placebo effect of an imaginary hypocaloric diet. Probably, some kind of psychological/motivational/behavioral therapy could become a very important part of the whole weight loss process. In our opinion, further studies on the placebo effect hypothesis in dieting are necessary in order more definitive conclusions to be derived. And second, regular physical activity of anaerobic–lactic type (performed in neutral energy balance condition) do not induce weight loss or changes in body composition in the short term. These findings are corroborated by many studies (38–40). In any case, the assessment of the potential effectiveness of a regular anaerobic physical activity on body mass and body composition changes in overweight and obese people requires further research. Additionally, we consider that our study only sets the basis for further investigations, which to reach to more decisive results and either replicate or repudiate ours.

## Ethics Statement

This study was carried out in accordance with the recommendations of the Scientific Projects Committee of Bulgarian National Sports Academy with written informed consent from all subjects. All subjects gave written informed consent in accordance with the Declaration of Helsinki. The protocol was approved by the Scientific Projects Committee of Bulgarian National Sports Academy.

## Author Contributions

VP organized the recruitment process and together with the dietitian held the initial interviews. The author managed the research process and was responsible for the statistical processing of the raw data. VP took part at the regular meetings of the participants and the dietitian and supervised the training sessions.

## Funding

This work was supported by the Scientific Projects Fund of the National Sports Academy of Bulgaria (Grant No. 223/09. 04. 2013). The terms of this arrangement have been reviewed and approved by the National Sports Academy of Bulgaria in accordance with its policy on objectivity in research.

## Conflict of Interest Statement

The author declares that the research was conducted in the absence of any commercial or financial relationships that could be construed as a potential conflict of interest.
